# Effects of high-fat diet and the anti-diabetic drug metformin on circulating GLP-1 and the relative number of intestinal L-cells

**DOI:** 10.1186/1758-5996-6-70

**Published:** 2014-06-02

**Authors:** Camilla Kappe, Qimin Zhang, Thomas Nyström, Åke Sjöholm

**Affiliations:** 1 Department of Clinical Science and Education, Karolinska Institutet, Unit for Diabetes Research, Södersjukhuset, SE-118 83 Stockholm, Sweden; 2 Department of Medical Cell Biology, Uppsala University, BMC, SE-751 23 Uppsala, Sweden; 3 Department of Internal Medicine, Södertälje Hospital, SE-152 86 Södertälje, Sweden; 4 Department of Biochemistry and Molecular Biology, University of South Alabama, College of Medicine, Mobile, AL 36688-0002, USA

**Keywords:** Metformin, Glucagon-like peptide-1, Insulinotropic, Lipotoxicity, L-cell

## Abstract

**Background:**

Elevated serum free fatty acids (FFAs) contribute to the pathogenesis of type-2-diabetes (T2D), and lipotoxicity is observed in many cell types. We recently showed that simulated hyperlipidemia induces lipoapoptosis also in GLP-1-secreting L-cells *in vitro*, while metformin confers lipoprotection.

The aim of this study was to determine if a high fat diet (HFD) reduces the number of enteroendocrine L-cells and/or GLP-1 plasma levels in a rodent model, and potential effects thereupon of metformin treatment.

**Methods:**

C57/Bl6 mice received control/HFD for 12-weeks, and oral administration of metformin/saline for the last 14 days. Blood glucose, glycosylated hemoglobin and plasma insulin and GLP-1 were determined before and after treatment with metformin using ELISAs. GLP-1-immunopositive cells in intestinal tissue sections were quantified using immunohistochemistry.

**Results:**

A HFD increased blood glucose, glycosylated hemoglobin, and fasting plasma insulin (33%, 15% and 70% increase, respectively), in conjunction with reduced oral glucose tolerance, indicating the manifestation of insulin resistance. Metformin counteracted these adverse effects, while also reducing prandial plasma FFAs. The number of GLP-1-positive cells was indicated to be reduced (55% reduction of the number of GLP-1-positive cells, *p =* 0.134), while there was a trend toward increased prandial plasma GLP-1 despite reduced food intake following a HFD.

**Conclusion:**

HFD-fed mice rapidly develop insulin resistance. Metformin exerts beneficial glucose lowering effects, and is indicated to improve the incretin response. Albeit no significant effect, a HFD tends to reduce the number of GLP-1-positive cells. However, considering concurrent normal or increased plasma GLP-1, any reduction in the number of GLP-1-positive cells, probably does not contribute to development of the glucose intolerance, but may contribute to progression of the diabetic state through eventual loss of a functional incretin response.

## Introduction

Glucagon-like peptide-1 (GLP-1) is an incretin hormone with insulinotropic effects, where its release in response to food intake stimulates insulin secretion in a glucose-dependent manner [1]. This incretin response has been suggested to be impaired in type 2 diabetes (T2D), characterized by hyperglycemia resulting from impaired insulin production and insulin resistance in peripheral tissues, as a result of reduced postprandial GLP-1 concentrations [2].

Administration of GLP-1 to patients restores glucose-induced insulin secretion as well as the β-cells’ sensitivity to glucose and normalizes fasting and postprandial glycemia in T2D patients [3], but its application as an antidiabetic drug is made difficult by rapid degradation. Native GLP-1 has a half-life of less than 2 minutes, due to degradation by dipeptidyl peptidase-4 (DPP-4) ubiquitously present in plasma. Stable analogs of GLP-1 and DPP-4-blocking agents that inhibit the degradation of active GLP-1 (7–36) amide to its metabolite GLP-1 (9–36) are available as treatments for T2D. Enhancing endogenous GLP-1 production/secretion by direct stimulation of GLP-1 secretion, and promotion of growth and viability, of L-cells may be a novel and more physiological option in incretin-based diabetes therapy. Enteroendocrine L-cells dispersed along the distal intestinal tract are the main source of endogenous GLP-1 secretion. Situated on the villi lining the intestine, the L-cells have apical microvilli facing the gut lumen and secretory vesicles located adjacent to the basolateral membrane. This morphology also enables the L-cells to be regulated by neuronal factors and hormones, as well as by direct nutrient stimulation [4,5]. Free fatty acids (FFAs) are known to be potent stimulators of GLP-1 secretion, as we and others have demonstrated *in vitro*[6]. FFAs also acutely stimulate GLP-1 secretion *in vivo*[7,8], where unsaturated fatty acids have proven more potent stimulators of incretin secretion than saturated ones [9].

Chronically elevated levels of circulating FFAs and hyperlipidemia, typically seen in T2D, are correlated to lipotoxicity and cellular dysfunction in many cell types. It has been reported that such lipotoxicity is induced when the capacity of the tissue for triglyceride storage is exceeded and the fatty acids are funneled towards other metabolic fates, such as β-oxidation and increased production of reactive oxygen species (ROS) [10]. In this context, it has also been shown that unsaturated fatty acids protect from saturated fatty acid-induced lipotoxicity through preventing increased β-oxidation and ROS production by promoting triglyceride storage of lipids [10]. This could be a result of increased solubility/stability of lipid droplets containing a higher percentage of unsaturated acyl chains [11].

Considering the link between obesity and T2D, reduced GLP-1 secretion in T2D, progressively diminished GLP-1 secretory response with increasing BMI [12], as well as the fact that high levels of FFAs induce apoptosis in a number of different cell types, we investigated if hyperlipidemia could result in increased apoptosis, and thus reduced GLP-1-positive cell number and GLP-1 secretory capacity - using an *in vitro* model. Our results [6,13] demonstrate that simulated hyperlipidemia does indeed induce apoptosis of GLP-1-secreting cells, through increased production of ROS. We also found that the most widely prescribed anti-diabetic drug, metformin, confers lipoprotection of GLP-1-secreting cells *in vitro*[6,13]. These results may provide an explanation to the increased levels of circulating GLP-1 in obese patients (with or without T2D) receiving metformin [14].

Leveraging the above findings, the aim of the present study was to determine whether a high fat diet (HFD) can reduce the number of enteroendocrine L-cells *in vivo* in a rodent model. In addition, we aimed to determine the effects of HFD treatment on GLP-1 plasma levels and possible effects of metformin on these parameters.

## Materials and methods

### Animals and diet

The use of laboratory animals was performed according to the guidelines of Karolinska Institutet, Sweden, in accordance with national law and approved by the local animal ethics committee (Stockholm South Animal Ethics Committee, permit # S30-12).

C57/Bl6 mice (Nova/Scanbur, Sollentuna, Sweden) arrived 9 weeks old and were housed in our animal department for 1 week prior to initiation of the study. Animals were housed in groups of four in a total of eight cages under standard laboratory conditions of light, temperature and humidity, and received food and water *ad libitum*. Metformin was purchased from Sigma-Aldrich, St. Louis, MO. At the initiation of the study, the animals were divided into two groups. One group received normal chow (Nova/Scanbur) and the other group received the fat-enriched D12492 diet consisting of 60% kcal% fat (Research Diets, New Brunswick, NJ). Since we aimed to determine not only the effects of a HFD, but also if such effects could be counteracted by metformin treatment, the animals receiving a HFD were divided into two groups (after 10 weeks of dietary treatment) - where eight animals received oral gavage administration of 300 mg/kg/day metformin and the remaining eight animals received oral administration of saline. Volumes administered ranged between 0.128 – 0.235 ml/day. After 14 consecutive days of metformin/saline administration -- 12 weeks after the initiation of the study -- animals were euthanized using carbon dioxide. Throughout the study, weight and food consumption for all animals/cages were recorded once a week.

### Blood glucose and HbA_1c_

Blood samples for glucose measurements were obtained from each mouse by needle puncture of the tail tip vein. Both prandial and fasting glycemia were measured at three time points (before initiation of metformin/saline treatment [time 0], 1 week after initiation [time 1] and 2 weeks after initiation before the end of the study [time 2]). Blood glucose concentrations were determined by means of Bayer’s Elite^®^ Glucometer and compatible blood glucose test strips. Fasting blood samples for HbA_1c_ measurements were obtained at time 0 and time 2 from the same puncture of the tail tip vein. HbA_1c_ was determined using a DCA Vantage^®^ Analyzer (Siemens Healthcare Diagnostics, Tarrytown, NY) and compatible sample cassettes.

### Serum insulin, FFA and GLP-1 determinations

Blood samples were collected from the saphena vein (approx. 50 μl – max. 8 ml/kg/2 weeks) after 6 h fasting at time 0, 1 and 2 (see above), and transferred to centrifuge tubes at room temperature. At time 2, immediately after euthanization, a heart puncture was also performed and a larger volume of prandial blood collected. The blood samples were spun to obtain serum. The serum was stored in a freezer at -70°C for later analysis of insulin, total GLP-1 (7–36 and 9–36), active GLP-1 (7–36) and FFAs. Serum insulin concentrations were determined using a mouse ultrasensitive enzyme-linked immunosorbent assay (ELISA) (Mercodia, Uppsala, Sweden), according to the manufacturer’s instructions. Serum GLP-1 (7–36 and 9–36) and serum GLP-1 (7–36) were determined using specific ELISAs (Millipore, Billerica, MA) according to the manufacturer’s instructions. Serum FFAs were determined using the NEFA-HR(2) assay kit (Wako Chemicals, Richmond, VA) according to its instructions.

### Oral glucose tolerance test (OGTT)

Food was removed 6 h prior to oral gavage administration of 1.5 g/kg glucose (50% glucose solution) at the end of the last week of treatment. At 15, 30 and 60 min following administration, blood samples for glucose measurements were obtained from each mouse by needle puncture of the tail tip vein. Blood glucose concentrations were determined by means of Bayer’s Elite^®^ Glucometer and compatible blood glucose test strips.

### Immunohistochemistry and quantification of L-cells

After euthanization, intestinal tissue was collected from the animals. The intestine was extracted and carefully removed of all fat. A 4-cm section proximal/distal to the appendix was removed and carefully cut open to have the lumen exposed and facing upward. The intestinal tissue section was then rolled up, starting with the most proximal end and with the lumen facing the center of the roll. The rolls were attached to plastic caps and immediately submerged in a freeze bath of 99% ethanol and dry ice. The rolls were stored in a freezer at -70°C pending sectioning of the intestinal tissue using a cryostat. The intestinal tissue was sectioned using a cryostat in 12 μm thick sections from the top of the roll towards the bottom, leaving each section to expose the lumen and villi of the full length of the intestinal tissue section. These sections were placed in consecutive order on six glass slides starting with position 1 of all slides, followed by position 2 of all slides, *etc*., leaving each and all slides with 10 sections representing the full cross section of the lumen. Sections were stored at -70°C.

Prior to IHC, sections were washed in PBS and fixed using a 10 min incubation with acetone at -20°C. Sections were then washed 3 × 10 min in PBS-T and incubated overnight at 4°C with a rabbit antibody specific for GLP-1 (Phoenix Europe GmbH, Karlsruhe, Germany. Cat# H-028-11) at 1:500 dilution in PBS-T containing 5% donkey serum. Tissue sections were washed in PBS-T 3 × 10 min and incubated for 2 h at room temperature in the dark with the secondary antibody (ALEXA Fluor 488 conjugated donkey anti-rabbit) (Invitrogen) at 1:200 dilution in PBS-T. Sections were washed in PBS 3 × 10 min and allowed to dry before a cover glass was mounted using Polyvinyl Alcohol mounting medium with DABCO antifading (Fluka Biochemica, Ronkonkoma, NY). For the purpose of quantifying cells after IHC, an advanced stereology platform, which allows unbiased quantification of cell number/volume and structural measurements within the intestinal structure [15], was used. Suboptimal quality of some of the pictures obtained from immunostaining of intestinal tissue resulted from technical difficulties associated with intestinal side effects of the combination of dietary treatment and metformin, as well as the relatively limited life-span of cryosections due to the formation of ice crystals Cross-sections from the distal intestine were analyzed from each of three animals from the different treatment groups.

### Statistical analysis

Comparisons between groups, treatments and time were made by a one-way ANOVA for repeated measures. Newman-Keul’s *post-hoc* test was used. Comparisons between control and single treatment groups were done using two-tailed Student’s *t* test. Correlations were evaluated by determining the Pearson correlation coefficients. *P <* 0.05 was deemed statistically significant. Power analysis was performed and taken into consideration for all experiments performed.

## Results

### After 10 weeks, C57/Bl6 mice on a high fat diet display a significant increase in body weight and a diabetic phenotype characterized by hyperglycemia and fasting hyperinsulinemia

**Figure 1:**
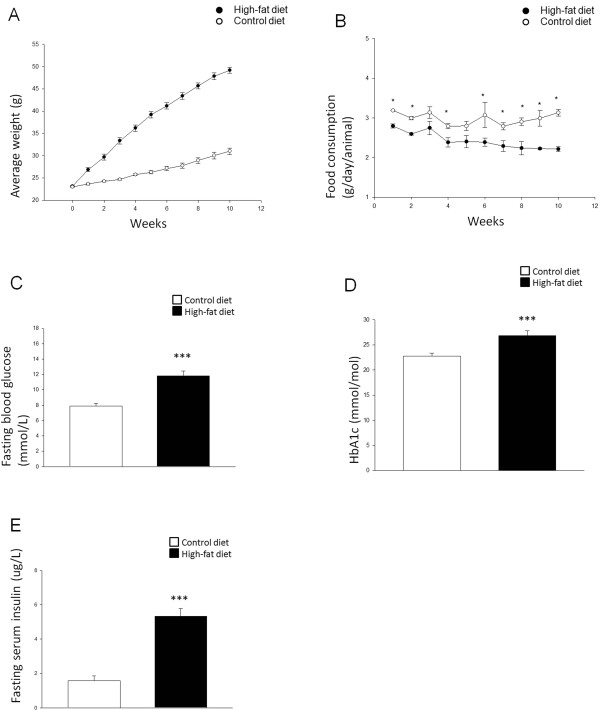
**After 10 weeks, C57/Bl6 mice on a high fat diet display a significant increase in body weight and a diabetic phenotype characterized by hyperglycemia and fasting hyperinsulinemia.** The average weight of the animals receiving a HFD is noticeably and significantly increased already after 1 week and continues to increase over 10 weeks **(A)***.* The average daily food intake **(B)** is slightly, but significantly, lower in the same group. Fasting blood glucose **(C)** and HbA_1c_**(D)**, as well as fasting insulin **(E)** levels were significantly higher in mice receiving HFD; 33%, 15% and 70% increase, respectively (n = 16 for control diet group, n = 16 for HFD group). Bars represent mean ± SEM. ***, *p <* 0.001 compared with control group. Statistical analysis was performed using Student’s t-test.

After 10 weeks on a HFD, mice had significantly increased in body weight (Figure 1A) while food intake was slightly -- but significantly -- reduced (Figure 1B). To determine if the HFD treatment had induced a diabetic phenotype, we analyzed glycemia, HbA_1c_ and fasting serum insulin levels. We could determine a significant increase in fasting blood glucose (Figure 1C), HbA_1c_ (Figure 1D) and fasting serum insulin levels (Figure 1E), 33%, 15% and 70% increase, respectively. In addition, serum was analyzed for the active form of GLP-1 (7–36) as well as total levels of GLP-1, including both the active peptide GLP-1 (7–36) and the metabolite GLP-1 (9–36). However, no difference in fasting active or total GLP-1 levels could be observed after 10 weeks on a HFD (control diet 22.1 ± 2.4 pM *vs.* HFD 23.0 ± 2.7 pM).

### Metformin significantly improves glycemia, fasting serum insulin and oral glucose tolerance

**Figure 2:**
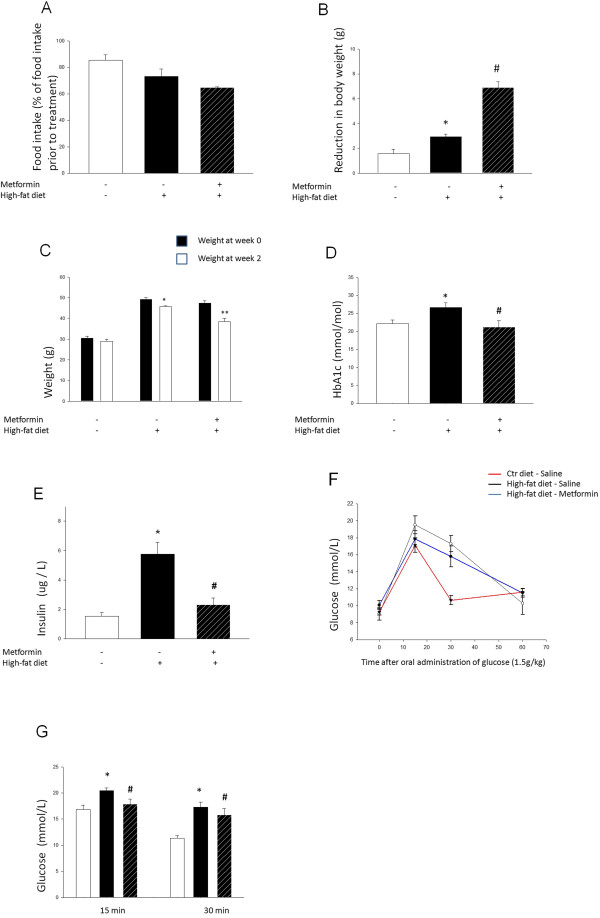
**Metformin significantly improves glycemia, fasting serum hyperinsulinemia and oral glucose tolerance.** Starting from week 1 of treatment, food intake was reduced in all groups but predominantly in the groups receiving HFD, where the metformin group displayed the largest decrease **(A)**. Due to the relatively short treatment period, and food intake being determined at only two time points (at the end of week 1 and week 2), statistical significance could not be calculated. The weight of the animals reflects the reduced food intake **(B, C)**, where the weight loss is greatest in animals receiving metformin in combination with HFD*.* HbA_1c_**(D)** and fasting serum insulin **(E)** levels are significantly increased in the HFD group, and significantly reduced (20% and 60% decrease, respectively) by a 14 day oral administration of 300 mg/day metformin. Glucose tolerance **(F,G)** is significantly decreased in mice receiving a HFD (37% increase in plasma glucose 15 min following oral glucose load), and significantly improved following a 14 day oral administration of 300 mg/day metformin; 6% reduction in plasma glucose (n = 8 for control diet group, n = 6 for HFD group, n = 4 for HFD group receiving metformin). Bars represent mean ± SEM. * and ** denote *p <* 0.05 and *p <* 0.01, respectively, compared with control group. #, *p <* 0.05 compared with HFD group. Statistical analysis was performed using one-way ANOVA.

Gauge feeding caused some nausea, reduced food intake and induced weight loss in all groups. The greatest reduction in food intake was observed in the groups receiving a HFD, where food intake was at its lowest among those receiving metformin in combination with the HFD. In conjunction with the data representing food intake, a relatively large additive weight loss was observed in the HFD + metformin group (Figure 2A-C). To determine the effect of metformin treatment on hyperglycemia and increased fasting serum insulin observed in mice on a HFD, these parameters were analyzed at the end of a two week treatment with metformin. An oral glucose tolerance test (OGTT) was also performed. Our results demonstrate that metformin significantly reduced HbA_1c_ (Figure 2D) and fasting serum insulin (Figure 2E) levels, *i.e.* a 20% and 60% decrease, respectively, was indicated. Considering the significant weight loss in HFD groups, we wanted to determine if weight could be an independent parameter regulating glycemia/serum insulin. Our data show a positive correlation between weight and HbA_1c_ (correlation coefficient: 0.683; *p <* 0.01). However, serum insulin levels cannot be correlated to weight alone. We also determined glucose tolerance using an OGTT. Glucose tolerance is significantly compromised in mice receiving a HFD as indicated by an elevated glucose peak (37% increase in plasma glucose 15 min following oral glucose load) and significantly higher glucose levels also 30 min after oral administration (Figure 2F-G). Nevertheless, daily oral administration of metformin for 14 days will significantly attenuate the glucose peak and blood glucose levels 30 min following oral administration (Figure 2G).

### A high fat diet significantly increases epididymal fat, serum free fatty acids and reduces the number of GLP-1-positive cells in the distal intestine while upregulating intestinal expression of GLP-1R mRNA

**Figure 3:**
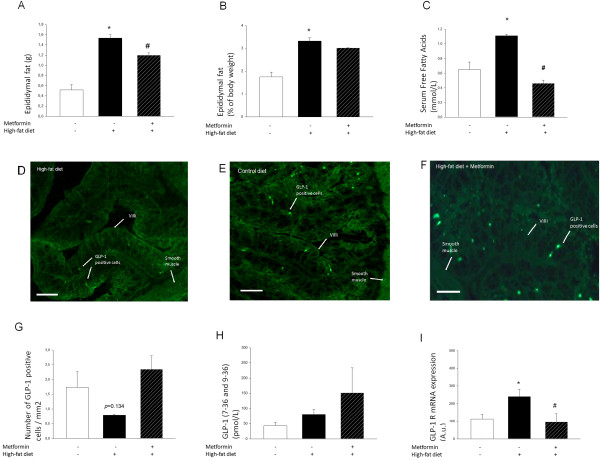
**A high fat diet significantly increases epididymal fat, serum free fatty acids and reduces the number of GLP-1-positive cells in the distal intestine*.*** Epididymal fat **(A-B)** and fasting serum free fatty acids **(C)** are significantly increased in animals receiving HFD (55% and 41% increase, respectively) and reduced by a 14-day 300 mg/day metformin treatment (22% and 59% reduction, respectively). (n = 8 for control diet group, n = 6 for HFD group, n = 4 for HFD group receiving metformin). There is a trend towards a reduced number of GLP-1-immunopositive cells in intestinal tissue sections from animals on HFD **(D and G)** as compared to animals receiving a control diet **(E and G)**; 55% reduction, *p =* 0.134, an effect indicated to be reversed by daily administration of metformin for 14 consecutive days **(F and G)**, (n = 3 for each treatment group). Scale bar: 25 μm. No significant effect can be determined of a HFD or metformin treatment on prandial serum GLP-1 (7–36 and 9–36) levels **(H)**; HFD - 46% increase, *p =* 0.095 and HFD + metformin – 72% increase, *p =* 0.23. Intestinal expression of GLP-1R mRNA was significantly upregulated in response to a HFD (53% increase), while normalized by metformin treatment. (n = 8 for control diet group, n = 6 for HFD group, n = 4 for HFD group receiving metformin). GLP-1R mRNA expression was normalized with mRNA expression of GAPDH, which was used as an internal control. A.u.; Arbitrary units **(I)**. Bars represent mean ± SEM. *, *p <* 0.05 compared with control group. #, *p <* 0.05 compared with HFD group. Statistical analysis was performed using one-way ANOVA.

Animals on a HFD had significantly more epididymal fat and prandial serum FFAs; however, these parameters were reduced in animals on a HFD also receiving metformin (Figure 3A-C). To determine if obesity and hyperlipidemia could induce a lipotoxic effect in these animals, as observed *in vitro*, resulting in reduced GLP-1-positive cell number and GLP-1 secretory capacity, we quantified GLP-1-positive cells in extracted intestinal tissue, using IHC and stereological methods. In line with a lipotoxic effect, we observed an indicated reduced number of GLP-1-positive cells in intestinal tissue sections from animals on a HFD (Figure 3D,G) as compared to animals receiving a control diet (Figure 3E,G). Specifically, a 55% reduction of the number of GLP-1-positive cells was indicated in the HFD group, with a p-value of 0.134. This effect was indicated to be reversed by daily administration of metformin for 14 consecutive days (f and g), However, there was not a significant difference between the two groups in terms of the intestinal expression of proglucagon mRNA (average mRNA expression after control diet 142 ± 26 *vs.* HFD 198 ± 73 [arbitrary units]). Further, fasting serum GLP-1 (7–36) levels were determined but indicated no significant difference between the groups (data not shown). As GLP-1 secretion is increased after nutrient intake, we also determined prandial GLP-1 (7–36 and 9–36) levels. Results from these studies indicate a trend toward increased prandial serum GLP-1 levels in animals receiving a HFD; however, this difference did not attain statistical significance. Further, treatment with metformin, while reducing epididymal fat and FFA levels, tends to enhance serum GLP-1 levels in animals receiving a HFD (Figure 3G). Further, in line with results from previous *in vitro* studies [6], metformin treatment reduced the intestinal expression of proglucagon (average mRNA expression after HFD-saline 198 ± 73 *vs.* HFD-metformin 49 ± 7 [arbitrary units]). Interestingly, intestinal expression of GLP-1R mRNA was significantly upregulated in response to a HFD, while normalized by metformin treatment (Figure 3H).

## Discussion

Animals on a HFD develop hyperglycemia in conjunction with hyperlipidemia, indicating the manifestation of insulin resistance. The improved glycemia, fasting serum insulin and oral glucose tolerance observed in response to metformin treatment was expected and in line with the known anti-diabetic properties of metformin [16,17]. The oral gavage administration did cause nausea and reduced food intake during the 14 days of treatment, where those mice receiving a HFD displayed significantly reduced appetite. Further, metformin treatment initially induced diarrhea. Starting with a lower dosage and increasing up to desired dosage over 3–4 days may have avoided this side-effect of metformin treatment. To detect if the weight loss during treatment was a confounding factor for the beneficial effects of metformin, we evaluated weight as an independent parameter for the improved metabolic state. The fact that weight alone displayed a significant positive correlation with HbA_1c_, but not fasting serum insulin, indicates that -- although the weight loss most likely contributed to the beneficial effects -- metformin treatment was indeed effective. In contrast to numerous reports of enhanced GLP-1 secretion in response to dietary fat [7,8], no significant increase in fasting or prandial GLP-1 (7–36 and/or 9–36) could be detected in our animals fed a HFD. This discrepancy may stem from differences in the fatty acid stimuli; lipid load/HFD, as well as the duration of the HFD. In fact, many of the before mentioned studies investigating effects of fatty acids on GLP-1 secretion have focused on acute effects, and it can be hypothesized that toxic effects of chronic hyperlipidemia and reduced L-cell mass at some stage outweigh the stimulatory effect of the enteral fatty acids on GLP-1 secretion, *i.e.* exceeding the ability of the remaining L-cells to compensate. Extending the duration of the HFD may thus eventually result in a defective postprandial GLP-1 response, while reducing the duration of the HFD may result in observations of increased plasma GLP-1 in this group, as was indeed recently demonstrated [18]. This hypothesis is supported by the trend toward reduced numbers of GLP-1-positive cells detected in the intestinal tissue from HFD-fed mice as compared to intestinal tissue from mice receiving a control diet, and in line with reports of a negative correlation between GLP-1 plasma levels and BMI [12]. Further, metformin was indicated to counteract such effects of a HFD on the number of GLP-1-positive cells. Detrimental effects only after persistent and long term exposure to hyperlipidemia could theoretically be explained by an accumulation of FFAs that eventually exceeds the capacity of the L-cell for triglyceride storage and the subsequent increase in β-oxidation and ROS production [10,13]. However, measurement of plasma GLP-1 in the different treatment groups following a glucose load would have added to the understanding of potential effects of a high fat diet and metformin treatment on the incretin response, and should be undertaken in future studies.

The present study shows reduced intestinal proglucagon expression in mice on a HFD following metformin treatment, which aligns well with previously published *in vitro* data, indicating metformin stimulatory action to be at the level of secretion. However, if metformin treatment increases the number of viable GLP-1-positive cells after a HFD, an increased intestinal proglucagon expression would be expected despite possible counteracting effects of metformin on proglucagon expression at the level of individual L-cells. The interpretation of the available data from this study is made even more difficult due to the adverse gastrointestinal side-effects induced by the metformin treatment, and the relatively short duration of metformin treatment. Therefore, further studies are necessary to confirm whether metformin lipoprotection can be observed *in vivo*, or is purely an *in vitro* phenomenon. The upregulation of GLP-1R mRNA in response to a HFD -- and normalization thereof by metformin treatment -- provokes further assessment of the intestinal expression of the GLP-1R under these conditions. Further, the mechanisms behind such effects remain to be investigated. It is, in light of a defective incretin response in diabetic patients improved by metformin treatment, tempting to hypothesize that compensatory mechanisms underlie increased receptor expression in response to reduced levels of the ligand and/or defective GLP-1R signaling in HFD-induced T2D.

In conclusion, our findings provide evidence for rapid development of insulin resistance and diabetes in mice receiving a HFD, with a significant improvement in response to metformin, which also was indicated to improve the prandial incretin response of HFD-fed mice. Further, our data demonstrate a clear trend toward reduced numbers of GLP-1-positive cells in HFD mice. However, considering no significant effect on fasting or prandial levels of GLP-1 in response to HFD, any reduction in the number of GLP-1-positive cells seemingly does not contribute to the development of oral glucose intolerance, hyperinsulinemia and hyperglycemia in this study. It may rather contribute to the progression of the diabetic state as it may lead to decreased prandial GLP-1 secretion when the reduction of GLP-1-positive cell number in response to hyperlipidemia overtakes fatty acid-induced potentiation of GLP-1 secretion.

## Abbreviations

GLP-1: Glucagon-like peptide-1; ROS: Reactive oxygen species; HFD: High fat diet; BMI: Body mass index.

## Competing interests

The authors declare that they have no competing interests.

## Authors’ contributions

CK carried out the studies, participated in the design of the study and drafted the manuscript. QZ participated in the design of the study and analysis and interpretation of data. TN participated in analysis and interpretation of data, and was involved in drafting the manuscript. ÅS conceived the study, participated in study design, analysis and interpretation of data as well as helped draft the manuscript. All authors read and approved the final manuscript.
